# Impact of molecular testing in clinical practice in gynecologic cancers

**DOI:** 10.1002/cam4.2064

**Published:** 2019-03-07

**Authors:** Marilyn Huang, Tegan Hunter, Brian Slomovitz, Matthew Schlumbrecht

**Affiliations:** ^1^ Department of Obstetrics & Gynecology, Division of Gynecologic Oncology, Sylvester Comprehensive Cancer Center University of Miami Miller School of Medicine Miami Florida; ^2^ University of Miami Miller School of Medicine Miami Florida

**Keywords:** cervical cancer, endometrial cancer, gynecologic cancers, Next generation sequence, ovarian cancer, personalized medicine, vulvar cancer

## Abstract

**Background:**

With the growing understanding of the molecular and genetic profiles of cancers, targeted treatments are increasingly utilized in personalized cancer care. The objective of this study was to determine how these advances have translated into practice by examining how often molecular profiling of gynecological tumors led to treatment changes.

**Methods:**

We identified women with gynecological cancers at our institution who had molecular tumor testing performed from November 2014 to June 2017. Clinicopathologic data were extracted from medical records. We determined (a) if molecular profiling identified actionable targets for which therapy is available, and (b) whether the patient's treatment course changed as a result of molecular profiling. Chi‐square, Wilcoxon rank‐sum, and Fisher's exact tests were used with a *P* < 0.05 considered statistically significant.

**Results:**

We identified 152 patients with gynecologic cancers who underwent molecular profiling. Of the 152 patients, 116 (76.3%) had actionable mutations identified, with 41 (35.3%) patients having a treatment change. Stratified by cancer type, molecular profiling most frequently identified an actionable target in patients with endometrial cancer (73.6%). Changes in treatment occurred most frequently in patients with endometrial cancer, 22 (56.4%), and ovarian cancers, 16 (39%), as compared to patients with cervical and vulvar cancer (*P* = 0.02). Of those patients who received a change in treatment, 39 patients (95.1%) received an FDA‐approved therapeutic agent, while two patients (4.8%) were enrolled in a clinical trial.

**Conclusion:**

Molecular profiling in gynecologic cancers often identified at least one actionable mutation; however, only in a minority of these cases was the course of treatment changed. Further studies are needed to elucidate optimal timing for testing to best utilize actionable information.

## 1 INTRODUCTION

An estimated 110 000 women are expected to be diagnosed with invasive gynecological cancer in the United States in 2018, which will lead to more than 32 000 deaths.^1^ Initial treatment for these cancers often involves a combination of surgery and chemotherapy. For those who present with advanced stage disease, and for whom surgery is not curative, survival varies by tumor type. In all gynecologic cancers, tumor recurrence after initial therapy is usually fatal.

When standard of care options fail, next generation sequencing (NGS) is increasingly utilized to identify molecularly targeted therapies to guide cancer treatments. Advances in NGS, increasing throughput and quality, have decreased time and cost involved with sequencing, allowing for more genes to be sequenced.^2^, ^3^ Identification of more gene alterations allows for increased use of targeted therapies. These potential therapies include hormone therapies, pathway‐specific therapies, and immunotherapies. NGS can identify opportunities for both FDA‐approved drugs as well as experimental therapeutics in clinical trials.

A retrospective study of 3727 cancer patients, with unrestricted site or stage of disease, identified actionable mutation or informative information in 73% of cases; only 19% of these results represented the standard of care.^4^ In a pilot study of patients with previously treated metastatic breast cancer, tumor molecular profiling resulted in a revision of the original treatment plan and a clinical benefit in 52% of the pretreated patients.^5^ In patients with non‐small cell lung cancer, NGS identified actionable mutations in 46% of the 209 patients for whom sequencing was performed; however, a targeted therapy was only instituted in 11% of sequenced patients.^6^ Finally, it has been shown that NGS‐guided therapy resulted in a longer progression‐free survival than unguided therapy. In a study of 1144 patients with multiple tumor types with both advanced and metastatic cancers, NGS‐guided therapy was associated with longer time to treatment failure and longer survival.^7^

Increasing evidence suggests that NGS has the potential to assist in clinical decision‐making and to increase the likelihood of response to targeted therapy in patients with recurrent cancers. However, it is unclear how NGS results are being implemented into clinical practice, and during the timeframe of the study, standard practice guidelines regarding timing and patient selection for NGS testing in gynecologic cancers were lacking. In March 2014, the Society of Gynecologic Oncology issued a clinical practice statement “Next Generation Cancer Gene Panels versus Gene by Gene Testing,” cautioning providers to consider both limitations and advantages by cancer gene panels.^8^ In February 2018, the most recent update of the National Comprehensive Cancer Network added the recommendation for tumor molecular testing prior to initiation of therapy for persistent/recurrent disease in ovarian cancer.^9^ However, there are no guidelines for genomic profiling in patients with other gynecologic cancers. Given the high cost of NGS testing, it is important that these results are being fully utilized. Our objective was to assess the frequency with which NGS identified clinically actionable mutations in patients with gynecologic cancers and to examine if such changes resulted in modification of prescribed therapies.

## 2 METHODS

We retrospectively identified patients diagnosed with gynecologic cancers and treated at our institution, who had molecular testing performed between November 2014 and June 2017. During this time period, NGS was increasingly used in our institution to screen gynecologic cancers for both the NCI MATCH study and to determine eligibility for other phase I trials. Patient data were extracted from the electronic medical record including demographic information, treatment history, and molecular testing results including next generation sequencing and protein expression with immunohistochemistry. Approval for this study was obtained from the University of Miami Institutional Review Board.

Demographic information was extracted from the electronic medical record and included patient age, race, ethnicity (Hispanic vs non‐Hispanic), personal history of cancer, any family history of cancer in a first‐degree relative (gynecologic and non‐gynecologic), and smoking status. Pathologic information obtained included stage, site of tumor, and histology at diagnosis.

Treatment history included information about neoadjuvant chemotherapy, surgical treatment, and adjuvant chemotherapy for the initial cancer diagnosis. Information on clinical trial participation and outcomes were abstracted. Treatment information was collected following recurrence including type of chemotherapy, if surgery was performed, the type of surgery, and outcome of any surgical interventions.

Tumor testing was performed on recurrent tumors obtained either by biopsy or following surgical resection. Molecular testing was performed using commercial services at the discretion of the treating physician from either Caris Life Sciences or Foundation Medicine. Caris Life Sciences tests for 639 genetic alterations and Foundation Medicine next‐generation sequencing detect alterations in 324 genes, including all 17 genes with FDA‐approved targeted therapies.

NGS results included potential actionable targets, for which there were either FDA‐approved treatments or investigational therapeutic trials. For the purposes of this study, an alteration was considered “actionable” when a therapeutic agent could be used to target that particular alteration. FDA‐approved treatments were listed on the NGS report, and investigational therapeutics were identified using the clinicaltrials.gov database. Investigators were unable to assess trial eligibility and other factors that affect enrollment such as location and cost, and therefore considered a mutation actionable without regard to eligibility. To determine if the molecular profiling results altered a patient's treatment, investigators assessed for treatment changes after results were received. If treatment was changed, investigators confirmed that the results supported this change. Data were collated from clinic note visits, telephone notes, and hospital admission records to elucidate rationale for not using NGS results (ie, change in patient performance status, response to another treatment, electing for clinical trial, or transitioning to hospice services).

Statistical analysis was performed using STATA1C 14 (StataCorp, College Station, Texas). Chi‐square (or Fisher's exact test, when appropriate) was performed to compare proportional differences. Wilcoxon rank‐sum test was used to compare nonparametric differences between groups of continuous variables. All tests were two‐sided, with a *P* < 0.05 considered statistically significant. For statistical analysis purposes, uterine sarcomas were included with the endometrial cancer cohort and vulvar cancers with the cervical cancer cohort.

## 3 RESULTS

There were 152 patients with recurrent gynecologic cancers who underwent molecular profiling between November 2014 and June 2017. We identified 78 patients with ovarian cancer (51.3%), 46 with endometrial cancer (30.3%), 15 with cervical cancer (9.8%), 6 with uterine sarcomas (3.9%), 5 with vulvar cancers (3.3%), and two patients with gestational trophoblastic disease (GTD) (1.3%). The majority of patients were white (73%) and non‐Hispanic (61%) with a median age of 57 years. The majority of patients (55%) had advanced stage disease (stage III or IV) at the time of diagnosis. In this cohort, 17.1% had a personal history of cancer, with breast cancer accounting for 38.5% of the cases. A summary of demographics is included in Table 1.

**Table 1:** Clinicodemographic comparison by actionable mutation status

Variable	Actionable mutation (%)	No actionable mutation (%)	*P*‐value
Median age, years (range)	57 (26‐81)	60 (14‐81)	0.28
Race			
White	64 (64.6)	35 (35.4)	0.31
Non‐White	21 (55.3)	17 (44.7)	
Ethnicity			
Non‐Hispanic	52 (57.1)	39 (42.9)	0.13
Hispanic	35 (70)	15 (30)	
Personal history of cancer			
No	74 (63.4)	42 (36.2)	0.74
Yes	20 (60.6)	13 (39.4)	
Family history of cancer			
No	21 (63.6)	12 (36.4)	0.86
Yes	65 (61.9)	40 (38.1)	
Smoking			
Never	63 (64.3)	35 (35.7)	0.67
Current/former	31 (60.8)	20 (39.2)	
Stage			
I/II	27 (69.2)	12 (30.8)	0.45
III/IV	51 (62.2)	31 (37.8)	
Site			
Non‐ovary	49 (67.1)	24 (32.9)	0.47
Ovary	41 (61.2)	26 (38.8)	
histology			
Non‐serous	65 (67.0)	32 (33.0)	0.11
Serous	25 (53.2)	22 (46.8)	
Histology			
Non‐endometrioid	75 (58.5)	53 (41.1)	0.004
Endometrioid	15 (93.8)	1 (6.2)	

The majority of patients did not receive neoadjuvant chemotherapy (76.3%) or maintenance therapy (92.0%) while most did receive adjuvant chemotherapy (85.7%) and surgery (91.3%). Of those patients whose disease status was known at the end of their primary therapy, 31% had progressive disease, 21% had a partial response, and 48% had a complete response prior to disease recurrence.

Of the 152 patients with recurrent gynecological disease, 116 (76.3%) had an actionable target identified, of which 41 (35.3%) had a subsequent change in treatment. When stratified by type of cancer, molecular profiling most frequently found an actionable target in patients with endometrial cancer (73.6%) followed by ovarian cancer (61.2%). In patients with vulvar or cervical cancer, 50% had actionable targets identified. When disease sites were evaluated specifically by proportion that displayed actionable mutations, there was no significant difference between sites (*P* = 0.13).

Of the 75 patients for whom NGS testing identified an actionable mutation, 28 (37.3%) patients were already receiving another form of treatment (chemotherapy or radiation) by the time NGS results were received. Ten patients (13.3%) elected to receive hospice or palliative care, and six (8.0%) experienced a decline in performance status and were unable to receive additional therapy. Six patients (8.0%) had no evidence of disease and required no additional chemotherapy. Three patients (4.0%) were deceased when results were received. The remaining patients were either lost to follow up, requested a transfer of care, or lacked detail in their chart to assess why treatment was not changed.

Among endometrial cancer patients, type I endometrial cancers had significantly more actionable mutations compared to type II endometrial cancers (93.8% vs 58.5%, *P* = 0.004). Furthermore, there was an increasing proportion of actionable mutations among endometrial/uterine cancer patients, though when compared to other disease sites, this was not statistically significant (Table 1). When evaluating patients with complete data on treatment changes by disease site, there was significant difference in proportion of patients with actionable mutations who had treatment changes due to NGS (n = 90). Changes in treatment occurred more frequently in patients with endometrial cancer (56.4%), and ovarian cancers (39%), as compared to patients with cervical and vulvar cancer (10%) (*P* = 0.02). All patients with endometrioid endometrial cancer had their treatments changed as a result of NGS testing compared with zero changes in patients with non‐endometrioid endometrial cancer (*P* < 0.001) (Table 2). In ovarian cancer, the most frequent actionable targets identified were ER (41%), PD1 (16.7%), and EFGR (11.5%), whereas in endometrial cancer, ER (58.7%), PTEN (58.7%), and CTNNB1 (19.6%) were more common. In cervical cancer, PIK3CA (33.3%), PD1 (26.7%), and EGFR (20%) were most common, whereas PD1 (40%) was most common in vulvar cancers (Table 3).

**Table 2:** Change in treatment plan by demographic and clinical factors (n = 94)

Variable	Change in treatment (%)	No change in treatment (%)	*P*‐value
Age			
<57 years	41.3	58.7	0.81
>=57 years	43.8	46.2	
Race			
White	45.3	54.7	0.56
Nonwhite	38.1	61.9	
Ethnicity			
Non‐Hispanic	40.4	42.9	0.82
Hispanic	59.6	57.1	
Personal history of cancer			
No	47.3	52.7	0.07
Yes	25	75	
Family history of cancer			
No	33.3	66.6	0.25
Yes	47.7	52.3	
Smoking			
Never	39.7	60.3	0.42
Current/former	48.4	51.6	
Stage			
I/II	52.3	47.7	0.06
III/IV	37.7	62.7	
Site			
Non‐ovary	46.9	53.1	0.45
Ovary	39.0	61.0	
Histology			
Non‐serous	46.2	53.8	0.38
Serous	36.0	64.0	
Histology			
Non‐endometrioid	0 (0)	51 (100)	<0.001
Endometrioid	39 (100)	(0)	

**Table 3:** Most frequently detected actionable mutations by cancer type

Cancer type	Most common mutations	Examples of targeted drugs
Ovary/fallopian tube and primary peritoneal	ER, BRCA 1, BRCA 2, PD1	Letrozole, PARP inhibitor, checkpoint inhibitor
Endometrium/uterine	ER, PTEN, CTNNB1	Tamoxifen, everolimus, temsirolimus, letrozole, medroxyprogesterone acetate
Cervix	PIK3CA, EGFR, PD1	Taselisib (clinical trial), gefitinib, checkpoint inhibitor
Vulva^a^	PD1	Checkpoint inhibitor

a Small number of cases

Overall, 41 patients received a change in treatment in response to NGS testing results. The most commonly chosen therapy was paclitaxel, with nine patients (21.9%). Eight patients (19.5%) were treated with doxorubicin, 4 (9.8%) with tamoxifen, 2 (4.8%) with olaparib, 2 (4.8%) with pembrolizumab, and 1 (2.4%) with pazopanib. Five patients (12.2%) were treated with everolimus and/or letrozole. One patient (2.4%) each enrolled in phase 1 clinical trials.

## 4 CONCLUSIONS

Advances in understanding of cancer biology and sequencing technology have facilitated the identification of “driver” alterations crucial in cancer propagation.^10^ Precision oncology is exemplified in breast cancer with the FDA‐approved *HER2*‐targeting agent because *HER2* amplification and overexpression are a proven predictive marker of response.^11^, ^12^ While the majority of molecular alterations do not have an FDA‐approved therapy, demand for improved or alternative therapies has driven next generation sequencing to map coding regions of cancer‐related genes. However, there is still much to be learned on the optimal timing of testing and incorporation into clinical practice. Intrapatient variability, with changes in the molecular profile from primary to metastatic disease and over the course of treatment, presents challenges to personalized medicine.^13^, ^14^, ^15^, ^16^ Furthermore, some authors have even attempted to classify potentially actionable alterations into categories with little success due to the rapidly evolving technology and knowledge.^17^ Published results vary widely depending on tissue type, but 39%‐83% are predicted to have a mutation for which matched therapy exists.^17^, ^18^

The rapidity in the evolution of molecular testing in tumors is demonstrated in one study examining a panel of mutations with SNP genotyping to identify 160 mutations across 15 cancer genes. During the study timeframe, genotyping was quickly replaced by broader next‐generation sequencing of 500‐1000 full gene sequencing.^19^ In the majority of our gynecologic cancer cases, molecular analysis identified an actionable target; however, only in a third of these cases was the course of treatment changed which is comparable to current literature in other disease sites.^20^

Our study identified that changes in treatment occurred most frequently in patients with endometrial/uterine cancers, followed by ovarian cancer compared to patients with cervical and vulvar cancer where few changes were made. In a recent study of 149 vulvar cancer cases, a variety of mutations were identified, with the following occurring most commonly: TP53 (33%), BRCA 2 (10%), HRAS (5%), FBXW7 (4%), and PIK3CA (3%). Targetable mutations by IHC included cMET (32%), PDL1 (18%), PTEN loss (56%), HER2 (4%), and ER (11%)/PR (4%).^21^ Many of the actionable targets had FDA‐approved drugs or clinical trials, which have potentially improved efficacy. Given that studies in other cancers have demonstrated that molecular profiling and targeting therapy can improve response and survival in cancer patients;^7^, ^22^ improved methods to utilize targeted therapy for patients are needed.

It is unclear whether the differences in treatment changes corresponding to NGS results by cancer type are due to the clinical availability and/or efficacy of additional standard treatments. There are by far, fewer clinical trials available for patients with vulvar cancer compared to ovarian, endometrial, and cervical cancers. In addition, we found that the most common reasons why treatment was not changed due to NGS results were that the patient had started another line of therapy or had a decline in performance status while waiting for NGS results. At the beginning of the study time period in 2014, receiving NGS testing results typically took 6‐8 weeks. It is possible that delays in testing and the length of time required to receive results meant that in many cases, results were received too late to influence therapy.

The optimal tissue specimen and timing of molecular profiling are controversial. Tumor specimens may be sent at any time during the patient's cancer diagnosis, including at initial diagnosis, at diagnosis of recurrence, and subsequent recurrences. For example, testing the original endometrial cancer specimen at the time of recurrence is unlikely to change the first‐line therapy recommendations or is it likely representative of the recurrent tumor mutational burden. Performing molecular profiling at the time of recurrence with a fresh biopsy and increasing the turnaround time could increase the number of patients who receive a targeted therapy.

In a retrospective analysis of 224 advanced stage ovarian cancer patients who underwent molecular profiling and subsequently received additional therapy, Herzog et al examined the value of tumor profiling on the survival of patients with advanced ovarian cancer.^23^ Patients were retrospectively divided into two cohorts based on whether or not the drugs they received matched their profile recommendations. The matched cohort received no drugs predicted to be lack‐of‐benefit while the unmatched cohort received at least one drug predicted to be lack‐of‐benefit. Profile biomarker and drug associations were based on multiple test platforms including immunohistochemistry, fluorescent in situ hybridization, and DNA sequencing. The matched cohort had a median OS of 36 months compared to 27 months for patients in the unmatched cohort (HR 0.62, 95% CI 0.41‐0.96; *P* < 0.03). The authors concluded that multiplatform molecular profiling may identify patients with ovarian cancer at risk of inferior survival and guide treatment selection based on results which may improve survival.^18^

Our finding that two patients (4.8%) enrolled in phase 1 clinical trials is consistent with results in other solid tumors that suggest providers are not enrolling patients for whom profiling identifies actionable mutations in clinical trials [3]. A study of 2000 consecutive advanced stage cancer patients found that NGS testing identified 789 patients (39%) with an actionable mutations, and of these patients, 83 (4%) went on to enroll in matched clinical trials targeting their mutation.^18^ Similarly, Stockly et al evaluated the frequency of genomic alterations, clinical “actionability” of somatic variants, enrollment in mutation‐targeted or other clinical trials, and outcome of molecular profiling for advanced solid tumors at a large cancer center. DNA from archival formalin‐fixed paraffin‐embedded tumor tissue was tested using a MADLI‐TOF MS hotspot panel or a targeted next generation sequencing panel. 1640 patients had testing performed, of which 245 patients (15%) were treated on therapeutic clinical trials, including 84 patients (5%) on genotype‐matched clinical trials. The authors concluded that few patients with advanced solid tumors were treated on genotype‐matched therapeutic trials.^22^

The National Cancer Institute Precision Medicine Initiative is an ongoing effort to conceptualize and test the feasibility of trials incorporating sequencing platforms and large‐scale bioinformatics processing that are not currently uniformly available to patients. This includes the National Cancer Institute‐Molecular Profiling‐based Assignment of Cancer Therapy (NCI‐MATCH), a nationwide hybrid trial that is designed to select treatment according to determined genetic alterations detected using next‐generation sequencing technology across a broad range of tumor types. Twenty‐four drug or drug combinations targeted to a specific molecular abnormality across the spectrum of cancer histologic subtypes were selected as 24 different therapeutic arms for evaluation in this trial. The primary end point of the NCI‐MATCH trial is overall response rate, and secondary outcomes included PFS of 6 months or greater, OS for each arm, and toxicity. After interim analysis, the screening goal for this trial was increased to 6000 patients from the initial 300 patients.^24^

Our study aimed to examine the utilization of molecular profiling in gynecologic cancers at a single institution. The strengths of this study are in its relatively diverse types of gynecologic cancers, having complete treatment records and the ability to determine if treatment decisions changed based on results. Limitations of the study include the small sample size in some groups and the retrospective nature of the study, which does not allow us to determine if targeted therapy guided by NGS extends patient survival, which would be better explored in a randomized controlled trial. Despite these limitations, however, we highlight that NGS data are not necessarily utilized to its full potential. While some of the underutilization is inevitably due to lack of clinical trials available for all patients, it is important to realize that in an era of cost‐containment, the goal of obtaining extra data on patient tumors should be to directly impact patient care.

In gynecologic cancers, specific actionable mutations were commonly seen; however, further studies are needed to guide usage of testing results to improve survival. If these specific mutations with effective therapies are identified, resources could be directed to ensure that these patients are treated with the corresponding agents. Finally, additional investigation is needed to determine why molecular profiling often does not lead to changes in treatment as this tool may improve survival.

## conflict of interest

The authors have no conflicts to disclose.

## **AUTHOR CONTRIBUTIONS**

Marilyn Huang involved in conceptualization, data curation, formal analysis, and writing—original draft, review, and editing. Tegan Hunter involved in data curation, formal analysis, writing—original draft, review, and editing. Brian Slomovitz involved in review, and editing. Matthew Schlumbrecht involved in formal analysis, writing, review, and editing.

## References

[1] Siegel RL , Miller KD , Jemal A . Cancer statistics, 2018. CA Cancer J Clin. 2018;68:7‐30. 29313949 10.3322/caac.21442

[2] Price KS , Svenson A , King E , et al. Inherited cancer in the age of next‐generation sequencing. Biol Res Nurs. 2018;20:192‐204. 29325452 10.1177/1099800417750746 PMC6030806

[3] Ulahannan D , Kovac MB , Mulholland PJ , et al. Technical and implementation issues in using next‐generation sequencing of cancers in clinical practice. Br J Cancer. 2013;109:827‐835. 23887607 10.1038/bjc.2013.416 PMC3749581

[4] Sholl LM , Do K , Shivdasani P , et al. Institutional implementation of clinical tumor profiling on an unselected cancer population. JCI Insight. 2016;1:e87062. 27882345 10.1172/jci.insight.87062 PMC5111542

[5] Jameson GS , Petricoin EF , Sachdev J , et al. A pilot study utilizing multi‐omic molecular profiling to find potential targets and select individualized treatments for patients with previously treated metastatic breast cancer. Breast Cancer Res Treat. 2014;147:579‐588. 25209003 10.1007/s10549-014-3117-1

[6] Hagemann IS , Devarakonda S , Lockwood CM , et al. Clinical next‐generation sequencing in patients with non‐small cell lung cancer. Cancer. 2015;121:631‐639. 25345567 10.1002/cncr.29089

[7] Tsimberidou AM , Iskander NG , Hong DS , et al. Personalized medicine in a phase I clinical trials program: the MD Anderson cancer center initiative. Clin Cancer Res. 2012;18:6373‐6383. 22966018 10.1158/1078-0432.CCR-12-1627 PMC4454458

[8] SGO Clinical Practice Statement . Next Generation Cancer Gene Panels Versus Gene by Gene Testing. 2014.

[9] Armstrong D , Plaxe SC , Alvarez , et al. NCCN Guidelines Ovarian Cancer. 2.2018 2018.

[10] Hanahan D , Weinberg RA . Hallmarks of cancer: the next generation. Cell. 2011;144:646‐674. 21376230 10.1016/j.cell.2011.02.013

[11] Smith I , Procter M , Gelber RD , et al. 2‐year follow‐up of trastuzumab after adjuvant chemotherapy in HER2‐positive breast cancer: a randomised controlled trial. Lancet. 2007;369:29‐36. 17208639 10.1016/S0140-6736(07)60028-2

[12] Slamon DJ , Leyland‐Jones B , Shak S , et al. Use of chemotherapy plus a monoclonal antibody against HER2 for metastatic breast cancer that overexpresses HER2. N Engl J Med. 2001;344:783‐792. 11248153 10.1056/NEJM200103153441101

[13] Gerlinger M , Rowan AJ , Horswell S , et al. Intratumor heterogeneity and branched evolution revealed by multiregion sequencing. N Engl J Med. 2012;366:883‐892. 22397650 10.1056/NEJMoa1113205 PMC4878653

[14] Swanton C . Intratumor heterogeneity: evolution through space and time. Cancer Res. 2012;72:4875‐4882. 23002210 10.1158/0008-5472.CAN-12-2217 PMC3712191

[15] Catenacci DV . Next‐generation clinical trials: novel strategies to address the challenge of tumor molecular heterogeneity. Mol Oncol. 2015;9:967‐996. 25557400 10.1016/j.molonc.2014.09.011 PMC4402102

[16] Bieg‐Bourne CC , Millis SZ , Piccioni DE , et al. Next‐generation sequencing in the clinical setting clarifies patient characteristics and potential actionability. Cancer Res. 2017;77:6313‐6320. 28939679 10.1158/0008-5472.CAN-17-1569 PMC5690871

[17] Johnson DB , Dahlman KH , Knol J , et al. Enabling a genetically informed approach to cancer medicine: a retrospective evaluation of the impact of comprehensive tumor profiling using a targeted next‐generation sequencing panel. Oncologist. 2014;19:616‐622. 24797823 10.1634/theoncologist.2014-0011 PMC4041676

[18] Meric‐Bernstam F , Brusco L , Shaw K , et al. Feasibility of large‐scale genomic testing to facilitate enrollment onto genomically matched clinical trials. J Clin Oncol. 2015;33:2753‐2762. 26014291 10.1200/JCO.2014.60.4165 PMC4550690

[19] Penson RT , Sales E , Sullivan L , et al. A SNaPshot of potentially personalized care: molecular diagnostics in gynecologic cancer. Gynecol Oncol. 2016;141:108‐112. 27016236 10.1016/j.ygyno.2016.02.032

[20] Hirshfield KM , Tolkunov D , Zhong H , et al. Clinical actionability of comprehensive genomic profiling for management of rare or refractory cancers. Oncologist. 2016;21:1315‐1325. 27566247 10.1634/theoncologist.2016-0049 PMC5189630

[21] Palisoul ML , Mullen MM , Feldman R , et al. Identification of molecular targets in vulvar cancers. Gynecol Oncol. 2017;146:305‐313. 28536037 10.1016/j.ygyno.2017.05.011

[22] Stockley TL , Oza AM , Berman HK , et al. Molecular profiling of advanced solid tumors and patient outcomes with genotype‐matched clinical trials: the Princess Margaret IMPACT/COMPACT trial. Genome Med. 2016;8:109. 27782854 10.1186/s13073-016-0364-2 PMC5078968

[23] Herzog TJ , Spetzler D , Xiao N , et al. Impact of molecular profiling on overall survival of patients with advanced ovarian cancer. Oncotarget. 2016;7:19840‐19849. 26942886 10.18632/oncotarget.7835 PMC4991422

[24] Coyne GO , Takebe N , Chen AP . Defining precision: the precision medicine initiative trials NCI‐MPACT and NCI‐MATCH. Curr Probl Cancer. 2017;41:182‐193. 28372823 10.1016/j.currproblcancer.2017.02.001

